# Status and perception toward the COVID‐19 vaccine: A cross‐sectional online survey among adult population of Bangladesh

**DOI:** 10.1002/hsr2.451

**Published:** 2021-12-14

**Authors:** Md Mostafizur Rahman, Musabber Ali Chisty, Mohammed Sadman Sakib, Masrur Abdul Quader, Ifta Alam Shobuj, Md Ashraful Alam, Md. Abdul Halim, Farzana Rahman

**Affiliations:** ^1^ Department of Disaster and Human Security Management, Faculty of Arts and Social Sciences Bangladesh University of Professionals Dhaka Bangladesh; ^2^ Institute of Disaster Management and Vulnerability Studies, University of Dhaka Dhaka Bangladesh; ^3^ Department of Global Health Policy, Graduate School of Medicine The University of Tokyo Tokyo Japan; ^4^ Department of Computer Science and Engineering Independent University Bangladesh Dhaka Bangladesh

**Keywords:** attitude, COVID‐19, hesitation, knowledge, vaccine

## Abstract

**Introduction:**

COVID‐19 has become a global public health concern. Safe and effective vaccines are required to control the pandemic. However, positive perception toward the vaccine is also necessary for a successful vaccination effort.

**Objective:**

A rapid online survey was conducted to evaluate the status and perception toward the newly administered COVID‐19 vaccine among the adult population (18 years and above) of Bangladesh.

**Methods:**

A total of 850 adult people participated. *χ* ^2^ or Fisher's exact test was performed to determine the association between the first dose of vaccination and sociodemographic information. Logistic regression analyses were carried out to examine the predictors of knowledge, attitude, and hesitation toward the vaccine.

**Results:**

Exactly 24.12% of the study population received their first dose of COVID‐19 vaccine, whereas 30.23% expressed hesitation about pursuing the vaccine. Older age groups (>70%), married people (49.62%), capital Dhaka city outsiders (32.76%), and high‐income groups (>50%) received the vaccine much higher than their counter group. Age, marital status, educational attainment, monthly income, and prior COVID‐19 positive status were all significantly associated with the knowledge regarding the vaccine. Only age (>55 years age group = aOR: 4.10; 95% CI: 1.30, 14.31) and level of knowledge (poor knowledge = aOR: 0.17; 95% CI: 0.12, 0.23) were significant determinants of attitudes. In case of hesitation, age group and monthly income were found as significant determinants. Fear of adverse consequences (86.67%) was the most common reason for hesitation, followed by insufficient information (73.85%).

**Conclusion:**

This study sought to determine the status and perception of the newly administered COVID‐19 vaccine to aid in the current inoculation campaign's effectiveness. Collaboration between academics, government officials, and communities is essential in developing a successful COVID‐19 vaccination program for the entire population. The authority should develop effective strategies to ensure the implementation of its policy of widespread COVID‐19 vaccination coverage.

## 1 INTRODUCTION

On March 11, 2020, the World Health Organization (WHO) declared the COVID‐19 pandemic, which was first detected in Wuhan, China.^1^, ^2^, ^3^, ^4^ As of April 7, 2021, over 131 million confirmed COVID‐19 cases worldwide, with over 2.86 million deaths.^5^ The situation has deteriorated significantly across the Americas, Europe, and Southeast Asia. India, Pakistan, and Bangladesh were the South‐Asian region's worst‐affected countries.^5^

Bangladesh reported the first COVID‐19 case on March 8, 2020.^6^ Afterward, the country observed a public holiday from March 26, 2020^7^ to reduce the outbreak. All educational institutions and offices were closed, but subsequently the offices reopened. The country has been battling this long‐term pandemic for years, notably in the socioeconomic and educational sectors.^8^, ^9^, ^10^, ^11^, ^12^ Maintaining social distance, wearing masks, and washing hands regularly proved challenging for the Bangladeshi people because of the highly crowded region, a large number of unconscious individuals, and a lack of knowledge about how to appropriately treat this pandemic.^9^, ^10^ Additionally, many individuals in the country were required to work outside to earn a living.^13^ Throughout the pandemic period, the capital Dhaka city became a hotspot and one of the worst‐affected COVID‐19 regions in the country.^9^, ^11^ In these circumstances, authorities faced impediments in promoting healthy behavior among the public.^14^, ^15^

People have been anticipating the development of an efficient COVID‐19 vaccine to avert the pandemic. Several vaccines have already been rollout.^16^ However, the success of the vaccination campaign is contingent on both its effectiveness and community acceptability.^17^ A positive impression of the vaccine is necessary to contain the outbreak.^18^ On January 27, 2021, the government of Bangladesh began administering the Oxford AstraZeneca vaccine Covidshield to the general population, and other vaccines like Moderna, Pfizer, and SinoPharma have also been launched (free of charge).^19^, ^20^ However, as is the case in many other countries, the government initially concentrated vaccination efforts on relatively particular categories of people (frontline medical personnel, government employees, private officials working on pandemic issues, and people of 40 years and over), with the entire population expected to be eligible for vaccination later.^20^, ^21^ 26 821 841 vaccines have already been administered, with 4.84% (7891624) of the entire population completely vaccinated as of September 2, 2021.^22^ However, there were growing safety concerns about the COVID‐19 vaccine in Bangladesh.^20^, ^23^ Several studies have already pointed out hesitation toward COVID‐19 vaccine.^18^, ^20^, ^24^, ^25^ These circumstances have made it critical to analyze the status and perception of the currently available COVID‐19 vaccine. The primary objective of this study was to determine the adult Bangladeshi population's status and perceptions regarding the new COVID‐19 vaccine. We intended to do so by comparing their vaccination status to associated sociodemographic factors. Additionally, we sought to ascertain their knowledge, attitudes, practices, and hesitations about COVID‐19 vaccination. To our knowledge, no prior research on the status, knowledge, attitude, and hesitancy toward the COVID‐19 vaccine has been published in Bangladesh.

## 2 MATERIALS AND METHODS

### 2.1 Study design and ethical issues

This cross‐sectional study was conducted in response to the Bangladesh government's decision to implement a nationwide COVID‐19 vaccination program.^26^ Due to the ongoing pandemic, a rapid self‐administered online survey was conducted to assess knowledge, attitude, practices, and hesitation regarding COVID‐19 vaccination among Bangladesh's adult population (18 years and older) with internet access. This study did not have more than minimal risk. It strictly followed all the associated ethical issues. After review, it has been approved by the Ethical Review Committee (Ref. ERC‐01/020221) of the Institute of Disaster Management and Vulnerability Studies, University of Dhaka, Bangladesh. Due to the rapid online survey, only online consent was taken. Online conversation and questionnaire's cover page described the objective and ethical issues of the study. Respondents could continue the survey if they agree with the information. No incentive was granted for participation.

### 2.2 Survey instrument and data collection

Literature reviews^9^, ^21^, ^27^, ^28^ were carried out to prepare the draft questionnaire (both in English and local Bengali language) in Google form. A pilot survey was conducted among some university students. Experts' opinion and cultural appropriateness were also considered. After the modifications, a final questionnaire was prepared. It had five major sections: general information contained sociodemographic information and previous COVID‐19 experience; knowledge section; attitude section; practice section; and hesitation section. The self‐administered online questionnaire was designed in such a way that respondents could receive different questions depending on their responses. If respondents reported receiving their first dose of the COVID‐19 vaccine, they were directed practices section toward COVID‐19 (to evaluate their preventive practices even after the first dose). However, when respondents indicated they had not yet received the vaccine, they were directed to an alternative question concerning their hesitations about the vaccine. If they reported hesitation, they were then directed to the final hesitation part. This part contained factors that could lead to hesitancy toward the vaccine. The final four sections (knowledge, attitude, practices, and hesitation) contained a total of 30 components. All the components were scored on 0 to 1 scale (correct/agree/hesitation = 1, neutral = 0.5, and wrong/do not know/disagree/no hesitation = 0). We believe that treating “do not know” as a 0 score was justifiable for our study.^29^ The knowledge section had 09 components with “true,” “false,” and “I do not know” responses. This section contained the vaccine's effectiveness, health behavior required to maintain even after the first dose of vaccination, side effects of the vaccine, and some misconceptions regarding the vaccine such as infertility and long‐term physical problems due to the vaccine. Five components were in the attitude section with agree, neutral, and disagree responses. Respondents reported their attitude regarding the pandemic, vaccine safety, effectiveness, and willingness for the vaccination. Nine good practices were only for the respondents who had already received the first dose of the vaccine. None of the respondents reported their second dose of the vaccine yet. The practice section contained healthy behavior such as using the mask, avoiding crowded places, and maintaining social distance. The hesitation section contained seven components regarding the COVID‐19 vaccine. The reported hesitation factors were ineligibility to pursue the vaccine, lack of confidence in the vaccine's ability to combat COVID‐19, lack of enough information, fear of side effects, cost, religious view, and other reasons. Additionally, we evaluated Cronbach's Alpha for reliability to assess the KAP section's internal consistency. Exactly 0.86, 0.80, and 0.73 were calculated for the knowledge, attitude, and practice section, respectively. The accepted value is >0.60.^30^, ^31^

A group of university students from the Bangladesh University of Professionals and the University of Dhaka, Bangladesh, were recruited for data collection. The selection was based on their research experience. They administered the study link from March 12 to April 2, 2021, through Facebook, email, WhatsApp, Imo, and Google classroom. The link was distributed to respondents who were easily accessible and competent in accessing the online survey. Thus, this study followed a non‐probability sampling technique. Even though an online‐based survey may not require a prior sample size, this perception‐based study estimated the sample size of 384 respondents (95% confidence interval [CI]) following Morgan's table.^32^ Approximately 980 respondents were approached. We received enormous responses where a total of 850 respondents participated. Thus, the overall response rate for the survey was 86.73%. Data were carefully monitored and double‐checked after each day of the data collection.

### 2.3 Data management and analysis

The collected data were transferred to Microsoft Excel in Office 365 (Redmond, Washington, USA).^33^ Afterward, data management and statistical analyses were performed in Python (version 2.7; Beaverton, OR 97008, USA) and “R” programming language (version 3.6.3; Vienna, Austria).^34^, ^35^ Descriptive statistics such as the frequency and percentages were calculated. The associations of sociodemographic information with the vaccination status were examined through *χ* ^2^ or Fisher's exact test, where appropriate. Post hoc analysis was carried out using the “rcompanion” package^36^ with the false discovery rate (fdr) method to compute the adjusted *P* value.

Overall scores (for the knowledge and attitude) were calculated by summing each section's component scores. Then, “Good” and “Poor” levels in the overall knowledge and attitude section were determined based on the 80% cutoff scores. Previous public health‐related research employed a cutoff score of 80% for good knowledge and attitude level.^37^, ^38^, ^39^ For the current study, 80% score in the overall knowledge section (overall score = 9) was calculated as 7.2. Seven and more than that was treated as “Good” knowledge level. A similar method was applied for the attitude section. Covariates were determined through *χ* ^2^ or Fisher's exact test. Univariate and multivariate logistic regression analyses were performed to determine the predictors of knowledge and attitude, where odds ratios (ORs), adjusted odds ratios (aORs), and 95% CI were estimated. Only the significant variables in the univariate analysis were considered in multivariate analysis. The logistic regression analyses were carried out to examine the predictors of hesitation toward the vaccine. Ninety‐five percent CI was considered for all statistical analyses.

## 3 RESULTS

Table 1 presents that 24.12% were vaccinated (first dose) in our study population. Age group, marital status, current accommodation, educational attainment, present occupation, monthly income, and previous COVID‐19 positive was significantly associated with the vaccination status. Post hoc analysis revealed that respondents aged 46 to 55 and over 55 were significantly more vaccinated than younger respondents. Individuals from Dhaka city were significantly less vaccinated (19.57%) than the Dhaka city outsiders (32.76%). University students were significantly less vaccinated (2.81%) than the business, employed, and unemployed individuals. Similarly, higher‐income respondents (30 000 BDT to 50 000 BDT) reported significantly more vaccinations (more than 50%) than lower‐income or “no income” respondents. Respondents who previously tested COVID‐19 positive vaccinated at a significantly higher proportion (34.80%) than those who did not (20.22%).

**TABLE 1:** First dose of COVID‐19 vaccination status

	Vaccinated (first dose)		
Features	n (%)	Yes (n (%))	No (n (%))
Total respondents	850 (100.00)	205 (24.12)	645 (75.88)
1. Age group (y)	***		
18 to 25	521 (61.29)	19 (3.65)	502 (96.35)
26 to 35	103 (12.12)	28 (27.18)	75 (72.82)
36 to 45	89 (10.47)	56 (62.92)	33 (37.08)
46 to 55	79 (9.29)	58 (73.42)	21 (26.58)
Above 55	58 (6.82)	44 (75.86)	14 (24.14)
2. Gender			
Male	446 (52.47)	107 (23.99)	339 (76.01)
Female	404 (47.53)	98 (24.26)	306 (75.74)
3. Marital status	***		
Married	262 (30.82)	130 (49.62)	132 (50.38)
Unmarried	521 (61.29)	28 (5.37)	493 (94.63)
Other (separated, divorced, widowed)	67 (7.88)	47 (70.15)	20 (29.85)
4. Living with family			
Yes	764 (89.88)	187 (24.48)	577 (75.52)
No	86 (10.12)	18 (20.93)	68 (79.07)
5. Current accommodation	***		
Dhaka city	557 (65.53)	109 (19.57)	448 (80.43)
Outside Dhaka city	293 (34.47)	96 (32.76)	197 (67.24)
6. Educational attainment	**		
Below higher secondary	40 (4.70)	18 (45.00)	22 (55.00)
Higher secondary	204 (24.00)	40 (19.61)	164 (80.39)
Above higher secondary	606 (71.29)	147 (24.26)	459 (75.74)
7. Present occupation	***		
Business	85 (10.00)	47 (55.29)	38 (44.71)
Employed	195 (22.94)	107 (54.87)	88 (45.13)
Unemployed	108 (12.70)	38 (35.18)	70 (64.81)
University students	462 (54.35)	13 (2.81)	449 (97.19)
8. Monthly income (BDT)	***		
No income	500 (58.82)	44 (8.80)	456 (91.20)
Below 15 000	74 (8.70)	06 (8.12)	68 (91.89)
15 000 to 29 999	37 (4.35)	07 (18.92)	30 (81.08)
30 000 to 49 999	83 (9.76)	42 (50.60)	41 (49.40)
Above 50 000	156 (18.35)	106 (67.95)	50 (32.05)
9. Previous COVID‐19 positive	***		
Yes	227 (26.70)	79 (34.80)	148 (65.20)
No	623 (73.29)	126 (20.22)	497 (79.78)

Abbreviation: BDT, Bangladeshi Taka.

** *P* < .01;

*** *P* < .001.

The further results (Table 2) also suggest that the majority of the study population reported positive responses regarding maintaining healthy behavior even after being vaccinated (88.82%), the vaccine has a potentiality to create some side effects (87.18%), concerns about the pandemic (87.06%). Exactly 86.24% of the respondents were aware of the website to register for the vaccination. It should be noteworthy that 59.41% of the study population reported the safety and effectiveness of the COVID‐19 vaccine, where only 46.94% were agreed that the vaccination could stop the pandemic. More than 35% of the respondents believed that the vaccine could create infertility and long‐term physical problems. More than 65% of them agreed to pursue the vaccine for themselves and their family members. Table 3 reports that the respondents were following the good practices after their first dose of the vaccine.

**TABLE 2:** Positive response (knowledge and attitude) regarding COVID‐19 vaccination (n = 850)

	Positive response	
Knowledge components	n (%)	95% CI
COVID‐19 vaccines are effective to prevent COVID‐19 infection	561 (66.00)	62.8 to 69.2
Need to maintain the health regulations after being vaccination	755 (88.82)	86.7 to 90.9
Vaccine will also help keep from getting seriously ill from COVID‐19	603 (70.94)	67.9 to 73.9
People being vaccinated can start to do normal activities	616 (72.47)	69.5 to 75.5
There is a website to register for COVID‐19 vaccination in Bangladesh	733 (86.24)	83.9 to 88.5
Vaccine has the potential for some side effects	741 (87.18)	84.9 to 89.4
Side effects due to the vaccination, normally go away in a few days	632 (74.35)	71.4 to 77.3
This vaccine can create infertility	531 (62.47)	59.2 to 65.7
This vaccine can create long‐term physical problems	519 (61.06)	57.8.8 to 64.3
**Attitude components**		
Concerned about the pandemic	740 (87.06)	84.8 to 89.3
This vaccine is safe and effective	505 (59.41)	56.1 to 62.7
If eligible, I need to take it as soon as possible	576 (67.76)	64.6 to 70.9
My family members and neighbors should take the vaccine, and I should be aware and motivate them to take it	562 (66.12)	62.9 to 69.3
Vaccination will help us to stop spreading COVID‐19	399 (46.94)	43.6 to 50.3

Abbreviation: CI, confidence intervals.

**TABLE 3:** Good practices after the first dose of COVID‐19 vaccine (n = 205)

	Good practices	
Components	n (%)	95% CI
Use mask when going outside	203 (99.02)	97.7 to 100.0
Follow trusted sources	202 (98.54)	96.9 to 100.0
Avoid crowded place	173 (84.39)	79.3 to 89.4
Wash hand	196 (95.61)	92.8 to 98.4
Wash clothes after return from outside	147 (71.71)	65.4 to 77.9
6 ft away from people	186 (90.73)	86.7 to 94.7
Cover mouth and nose when I cough or sneeze and then dispose the tissue and wash hands immediately	187 (91.22)	87.3 to 95.1
Wear masks even with a vaccinated person	167 (81.46)	76.1 to 86.8
Visit hospital when symptoms of COVID‐19 appear	168 (81.95)	76.6 to 87.3

Abbreviation: CI, confidence intervals.

Exactly 62.12% and 68.18% of individuals reported good knowledge and good attitudes toward the COVID‐19 vaccine. Univariate logistic regression analyses (Table 4) determine the age group, marital status, educational attainment, present occupation, monthly income, and previous COVID‐19 positive as significant predictors of knowledge regarding the COVID‐19 vaccine. The 46 to 55 years and above 55 years age group reported that increased odds of having a good knowledge (OR: 4.48; 95% CI: 2.41, 9.14 and OR: 4.53; 95% CI: 2.22, 10.51 respectively) compared with the youngest age group (18‐25 years). In contrast, decreased odds of having a good knowledge (OR: 0.52; 95% CI: 0.38, 0.71 and OR: 0.33; 95% CI: 0.16, 0.62) were found when the study population were unmarried and less educated compared with the married and more educated, respectively. Respondents who had business and earned more than 50 000 BDT/month reported an increased odds of having a good knowledge; (OR: 2.14; 95% CI: 01.29, 3.71) and (OR: 01.93; 95% CI: 1.30, 2.94) compared with the university students and “no income” respondents, respectively. Individuals who tested COVID‐19 positive before showed increased odds of having a good knowledge (OR: 1.91; 95% CI: 1.37, 2.67) compared with those who did not. In the case of multivariate analysis, all these factors remained significant predictors of knowledge except present occupation. Older age group (46‐55 years and above 55 years) and previous COVID‐19 positive individuals showed increased odds of having a good knowledge; (aOR: 3.80; 95% CI: 1.46, 10.44), (aOR: 5.05; 95% CI: 1.67, 16.87) and (aOR: 1.69; 95% CI: 1.18, 2.43), respectively. In contrast, decreased odds of having a good knowledge was found in case of higher secondary (aOR: 0.65; 95% CI: 0.45, 0.92) and below secondary (aOR: 0.12; 95% CI: 0.05, 0.27) educational attainment, and below 15 000 BDT/monthly earned (aOR: 0.40; 95% CI: 0.23, 0.69) respondents. Univariate analyses determine the age group, marital status, present occupation, and knowledge level as significant predictors of attitude. Above 55 years age group and employed participants reported higher odds of good attitude; (OR: 3.98; 95% CI: 1.89, 9.77) and (OR: 1.73; 95% CI: 1.19, 2.55) compared with the 18 to 25 age group and university students, respectively. Lower odds of good attitude were observed when unmarried (OR: 0.67; 95% CI: 0.48, 0.92) and poor knowledge (OR: 0.17; 95% CI: 0.12, 0.23) were compared with the married and good knowledge, respectively. Multivariate analyses identified only age group and knowledge level as significant predictors of attitude. Above 55 years age group demonstrated increased odds of having a good attitude (aOR: 4.10; 95% CI: 1.30, 14.31), whereas poor knowledge reported lower odds of good attitude (aOR: 0.17; 95% CI: 0.12, 0.23).

**TABLE 4:** Significant predictors of knowledge and attitude regarding COVID‐19 vaccination (n = 850)

	Knowledge		Attitude	
Predictors	OR (95% CI)	aOR (95% CI)	OR (95% CI)	aOR (95% CI)
Age group (y)				
18 to 25	1		1	
26 to 35	0.74 (0.48, 1.13)	0.65 (0.32, 1.28)	0.97 (0.63, 1.52)	1.01 (0.48, 2.11)
36 to 45	1.23 (0.78, 1.97)	0.93 (0.42, 2.05)	1.48 (0.941, 2.48)	1.73 (0.74, 4.05)
46 to 55	4.48 (2.41, 9.14)***	3.80 (1.46, 10.44)**	1.72 (1.02, 3.05)	1.55 (0.62, 3.95)
Above 55	4.53 (2.22, 10.51)***	5.05 (1.67, 16.87)**	3.98 (1.89, 9.77)***	4.10 (1.30, 14.31)*
Marital status				
Married	1		1	
Unmarried	0.52 (0.38, 0.71)***	0.54 (0.32, 0.91)*	0.67 (0.48, 0.92)*	
Other (separated, divorced, widowed)	1.57 (0.84, 3.10)	0.95 (0.42, 2.19)	1.07 (0.59, 2.03)	
Educational attainment				
Above higher secondary	1			
Higher secondary	0.79 (0.57 to 1.10)	0.65 (0.45, 0.92)*		
Below higher secondary	0.33 (0.16, 0.62)***	0.12 (0.05, 0.27)***		
Present occupation				
University students	1		1	
Business	2.14 (1.29, 3.71)**		0.91 (0.57, 1.48)	
Employed	1.28 (0.91, .82)		1.73 (1.19, 2.55)**	
Unemployed	1.15 (0.75, 1.78)		1.57 (0.99, 2.55)	
Monthly income (BDT)				
No income	1			
Below 15 000	0.36 (0.21, 0.59)***	0.40 (0.23, 0.69)**		
15 000 to 29 999	0.73 (0.37, 1.44)	1.22 (0.40, 4.02)		
30 000 to 49 999	1.22 (0.76, 2.02)	1.51 (0.50, 4.92)		
Above 50 000	1.93 (1.30, 2.94)***	1.69 (1.18, 4.17)		
Previous COVID‐19 positive				
No	1			
Yes	1.91 (1.37, 2.67)***	1.69 (1.18, 2.43)**		
Knowledge				
Good			1	
Poor			0.17 (0.12, 0.23)***	0.17 (0.12, 23)***

Abbreviation: aOR, adjusted odds ratio; BDT, Bangladeshi Taka; OR, odds ratio.

* *P* < .05;

** *P* < .01;

*** *P* < .001.

**FIGURE 1:**
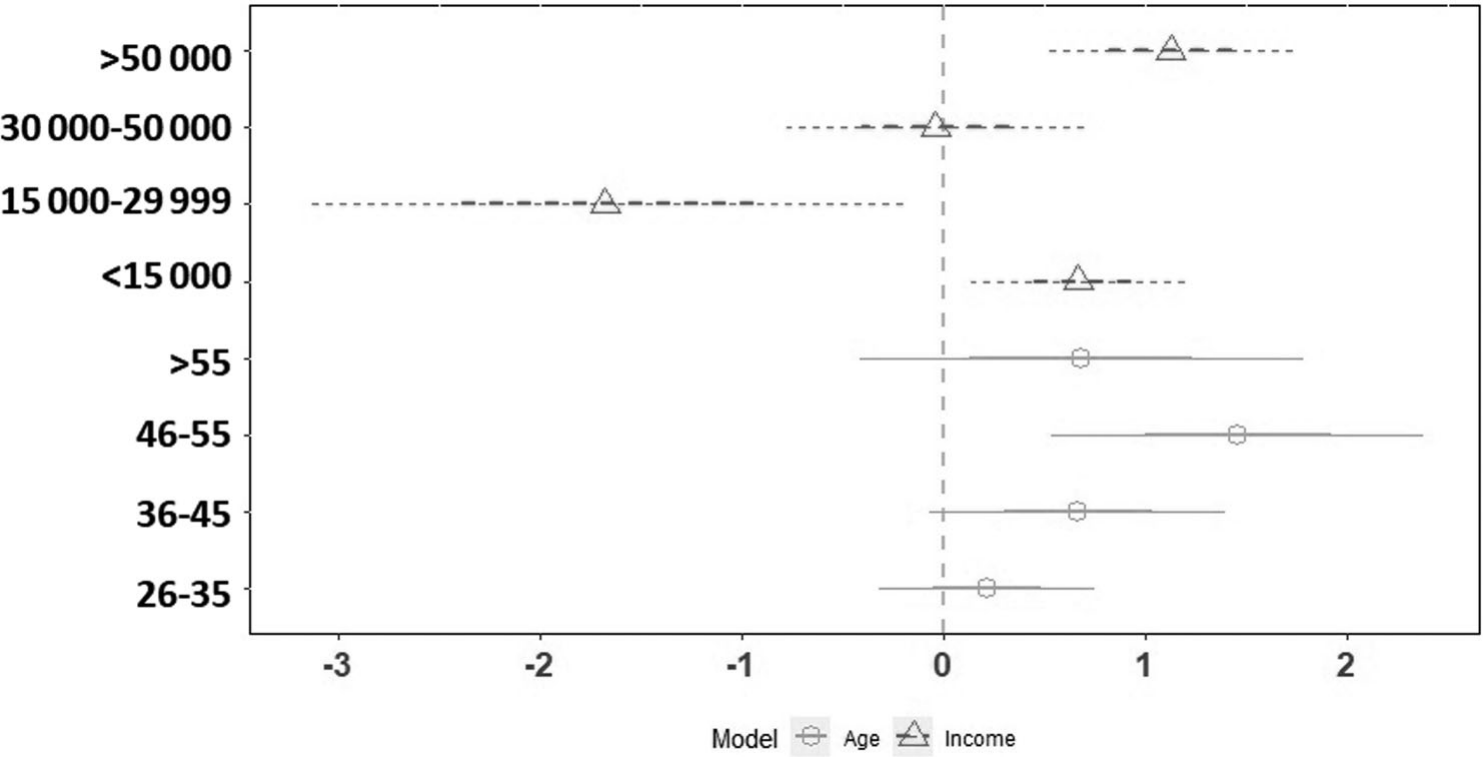
Significant predictors of hesitation regarding COVID‐19 vaccination (n = 645)

Exactly 30.23% of individuals, who did not receive the vaccine yet, reported their hesitation to pursue the vaccine. Figure 1 shows that age and income are significant predictors of hesitation toward COVID‐19 vaccination (n = 645). Points (circles and triangles) denote coefficient for age group and income, respectively. Lines extending from the circles represent the 95% CI. Points showing in the dashed line's right indicate an increased hesitation to pursue the vaccine, whereas points on the left of the dashed line indicate decreased hesitation toward the vaccine. CI lines without touching the dashed line indicate a significant value. The 46 to 55 age group, with a monthly income of less than 15 000 BDT and above 50 000 BDT, reported increased odds of having hesitation toward the vaccine. In contrast, a monthly income of 15 000 to 29 999 BDT reported decreased odds of having hesitation regarding the vaccine.

We also investigated the factors triggering the hesitation toward COVID‐19 vaccination (Table 5). Afraid of side effects was reported as the top hesitation factor (86.67%), followed by the lack of enough information (73.85%).

**TABLE 5:** Factors leading to hesitation toward COVID‐19 vaccination (n = 195)

	Hesitated	
Reasons	n (%)	95% CI
Might be ineligible for the vaccine	71 (36.41)	29.6 to 43.2
Lack of confidence on COVID‐19 vaccine to combat COVID‐19	85 (43.59)	36.6 to 50.6
Lack of enough information	144 (73.85)	67.6 to 80.0
Afraid of side effects	169 (86.67)	81.8 to 91.5
There might be cost for the vaccine	22 (11.28)	6.8 to 15.8
Might be against my religious belief	13 (6.67)	3.1 to 10.2
Other reasons (not to mention)	107 (54.87)	47.8 to 61.9

## 4 DISCUSSION

COVID‐19 has developed into a global health threat, and the world is awaiting an effective vaccine to avert this situation.^40^, ^41^, ^42^ International communities have considered vaccines as a means of ensuring public safety.^43^ However, a lack of knowledge, a negative attitude, and increased hesitancy may cause the vaccination to be delayed. WHO has labeled vaccine hesitancy as one of the top 10 global health threats in 2019.^44^ Concerns about the vaccine's efficacy and safety against new COVID‐19 variants have also grown.^45^, ^46^

We found that the older age group in Bangladesh were vaccinated more than the younger age group. This result is consistent with the government's strategy, under which only people aged 40 and over were eligible for the vaccine.^47^ Frontline health workers and other high‐risk government and private sector employees have received the vaccine before reaching this age limit. Additionally, the government announced the commencement of a vaccination campaign for university students and staff.^48^ The primary objective of this campaign was to reopen educational institutions after ensuring a low risk of infection. During this study's analyses (April 4, 2021), a rapid trend was observed in COVID‐19 cases among the younger group. The sudden spike of COVID‐19 cases^49^ may result in an increase in the number of people vaccinated. Our study found that capital city residents were vaccinated at a lower rate than city outsiders. As of June 13, 2021, about 58.9% of total COVID‐19 cases were reported in Dhaka city.^50^ Even though authorities have already planned mass vaccinations with a focus on the worst‐affected capital city,^51^ this densely populated area should continue receiving coverage. Monthly income and previous COVID‐19 positive were also found associated factors with the first dose of vaccination. Individuals who have already been infected may seek more protection from the disease. As a result, they may be more receptive to vaccination than those who have not tested positive previously.

Additionally, our findings indicate that Bangladeshis require vaccine‐related public awareness campaigns to dispel vaccine myths and misconceptions. The majority of the population knew the website for the vaccination in Bangladesh. Bangladesh government launched a website where people can register online^21^ to pursue the vaccine. However, it may be challenging for those who lack internet access or are technologically challenged and uninterested. Bangladesh's government also launched a walk‐in vaccination campaign to cover these people, but it was suspended due to additional difficulties, such as mismanagement.^51^, ^52^ Most of the study population were aware of the health behaviour to maintain even after the first dose of vaccination. Less than half of this population agreed that the vaccine could stop the pandemic. A previous study indicated that the vaccine has the potential to boost herd immunity and thus stop the pandemic.^18^ Many participants of the study population were skeptical about the vaccine's safety and effectiveness. This finding underscores the crucial need for authentic knowledge dissemination regarding the safety and efficacy of the COVID‐19 vaccine.

We also found that the older age group demonstrated better knowledge and attitude regarding the vaccine than the youngest group. A high proportion of this group's population being already vaccinated may contribute to this positive attitude toward the vaccine. Government campaigns targeting this group may also raise their awareness of the vaccine. Nonetheless, they were the pandemic's most vulnerable group, which may have sparked their interest in vaccination‐related information. Additionally, our findings suggest that the individuals' prior COVID‐19 infection steered them toward good knowledge. The results identified education as essential factor to improve the perception of the vaccine. It also corresponds to another study,^9^ which considered response toward the COVID‐19 pandemic. This study observes the positive association between the knowledge level and attitude level. It demonstrates that increased knowledge distribution can result in more favorable attitudes toward the vaccine. Additionally, this study's findings indicate that married individuals are more aware of the vaccine than unmarried individuals. A married individual may be concerned about their spouse. The vaccine can reduce the risk of infection for both. Besides, knowledge distribution can be well enough when they discuss with their partner. Education was also found to be an essential associated factor in improving the knowledge level. Around 70% of the study population (who had not yet received the vaccine) expressed an intention to pursue the vaccine. Age and income were found only the associated factors with the hesitation. The factors that contributed to the reluctance of the remaining members of the unvaccinated group indicate the critical need for a comprehensive vaccination campaign.

A holistic approach must be considered to accelerate the safe and effective COVID‐19 vaccine. The authority should adopt a strategy in which all sectors, including government and private organizations, local governments, educational institutions, health professionals, disaster management practitioners, and community leaders, can ensure adequate awareness regarding the vaccine. Along with regular COVID‐19 status monitoring, governing authorities should organize a series of campaigns, social mobilization, and communication regarding the successful inoculation campaign. Online campaigns utilizing web‐based and mobile applications may potentially be useful in light of the ongoing COVID‐19 pandemic. Television and social media platforms may also be used to disseminate the awareness programs such as short documentaries, dramas, and case studies on COVID‐19 vaccination at the community level. Social media has grown in importance as a source of information for the public in Bangladesh. However, all these techniques must take internet access, literacy, and cognitive comprehension into account when designed and implemented. Interacting with community leaders, posters, miking, and mobile messaging may be useful for disseminating accurate information. Health and disaster management agencies may potentially use these systems for pandemic‐related information. Authority should increase the community‐based clinic and vaccination booth for the online registration and vaccination. Walk‐in vaccination campaigns may also be reconsidered with proper management. They can incorporate more staff to manage the whole process effectively. The authorities must equip and train their employees and other key stakeholders to battle this contagious disease. Additionally, a significant collaboration between academics, authorities, and communities is necessary to develop a successful COVID‐19 vaccination campaign for all people. The authority should use all these strategies to carry out its policy of comprehensive COVID‐19 vaccination coverage.

Our effort is to assess the perception of the new COVID‐19 vaccine among the adult population of Bangladesh. There are some limitations to the current study that should be considered when interpreting the findings. It used non‐probability sampling techniques to conduct an online questionnaire survey in response to the ongoing COVID‐19 pandemic. As a result, this survey may contain some biases. For instance, respondents may consider socially acceptable responses even when the survey pattern is anonymized. Additionally, they required access to the internet to participate in the survey. The majority of them reported having completed at least higher secondary. They might already have better knowledge regarding the COVID‐19 vaccine. The findings were based solely on self‐reported online survey responses without additional validation. For example, respondents indicated that they had previously been infected with COVID‐19, but due to the anonymous online survey pattern, it was not confirmed by their medical records. Despite these limitations, this exploratory study may still provide essential information to Bangladeshi authorities and assist other COVID‐19‐affected communities in conducting effective vaccination campaigns.

## 5 CONCLUSIONS

COVID‐19 has become a long‐term burden for many countries. Along with the non‐therapeutic treatments such as the following health behavior, a long‐awaited effective vaccine can demolish this burden. However, positive perceptions without hesitation regarding the vaccine can only make the whole process successful. This study revealed the requirement of an extensive campaign to motivate the Bangladeshi people toward the vaccine. More people should be covered without considering the sociodemographic information to ensure herd immunity. A holistic approach is required where people from multisectoral fields should be incorporated.

## CONFLICT OF INTEREST

The authors declare that they have no conflict of interest.

## AUTHOR CONTRIBUTIONS

Conceptualization: Md Mostafizur Rahman, Musabber Ali Chisty, Mohammed Sakib, Masrur Quader, Ifta Shobuj, Md Alam, Md Halim, Farzana Rahman

Data curation: Md Mostafizur Rahman, Mohammed Sakib, Masrur Quader, Ifta Shobuj

Formal analysis: Md Mostafizur Rahman

Investigation: Md Mostafizur Rahman, Musabber Ali Chisty

Methodology: Md Mostafizur Rahman, Musabber Ali Chisty

Resources: Md Mostafizur Rahman, Musabber Ali Chisty

Software: Md Mostafizur Rahman

Supervision: Md Mostafizur Rahman

Writing—Original Draft: Md Mostafizur Rahman, Musabber Ali Chisty, Mohammed Sakib, Masrur Quader, Ifta Shobuj, Md Alam, Md Halim, Farzana Rahman

Writing—Review and Editing: Md Mostafizur Rahman, Md Alam

All authors reviewed, discussed, and agreed to the final version of the manuscript.

## TRANSPARENCY STATEMENT

Md Mostafizur Rahman affirms that this manuscript is an honest, accurate, and transparent account of the study being reported, that no important aspects of the study have been omitted, and that any discrepancies from the study as planned have been explained.
